# Transcription-coupled repair of crosslinking DNA damage

**DOI:** 10.1016/j.dnarep.2025.103869

**Published:** 2025-07-16

**Authors:** Rebecca Roddan, Lucy R. Henderson, Malitha Ratnaweera, Peter J. McHugh

**Affiliations:** Department of Oncology, MRC Weatherall Institute of Molecular Medicine, University of Oxford, Oxford OX3 9DS, UK

**Keywords:** DNA crosslinks, Interstrand crosslinks, DNA protein crosslinks, Excision repair

## Abstract

Impediments to faithful transcription must be resolved to ensure accurate gene expression and safeguard normal cellular function. Dedicated DNA repair pathways have therefore evolved to remove transcription-blocking DNA damage, targeted to active genes. Although significant research efforts to date have focussed on the transcription-coupled repair of bulky, UV-induced DNA damage, it is known that other forms of DNA damage can perturb RNA Polymerase II progression. Only in recent years has insight into these pathways emerged, despite the clinical significance of understanding all transcription-coupled repair pathways. These recent observations have highlighted substantial molecular differences in these pathways compared to the canonical UV-damage repair mechanisms. This review summarises our understanding to date of the molecular mechanisms that act to remove both DNA-DNA and DNA-protein crosslinks that block transcription in mammalian cells.

## 1 Introduction to transcription-coupled repair

Studies elucidating the molecular details underlying rare genetic diseases have been fundamental to the field of DNA repair. By correlating phenotype to genotype, researchers have been able to identify DNA repair factors and the pathways in which they operate [1]. For example, key details of nucleotide excision repair (NER) resulted from the study of Xeroderma pigmentosum (XP), a rare genetic disorder characterised by sunlight sensitivity and cancer predisposition. XP patients have defects in genes encoding for nucleotide excision repair (NER) factors, which act to remove bulky DNA lesions induced by sunlight. It was found in experiments using XP patient fibroblasts that (i) NER could still occur when repair factor XPC was mutated, but that this repair was confined to active genes [2,3] and (ii) RNA synthesis was decreased after UV irradiation [4]. These observations helped delineate transcription-coupled NER (TC-NER) from the global genome repair (GGR) of UV-induced intrastrand crosslink lesions.

In the mid-1980s, pioneering work from the Hanawalt group demonstrated that UV-induced lesions in actively transcribed genes are repaired faster than the overall genome in Chinese Hamster ovary (CHO) cells, reflecting enhanced repair of the transcribed strand [5,6]. Complementary work also demonstrated that TC-NER occurs in all kingdoms of life, albeit through distinct mechanisms, and a historical perspective of these early studies is available [7]. Prior studies of another rare disease, Cockayne Syndrome (CS), provided potential key insights into the genetic basis of what became known as transcription-coupled repair (TCR) [4]. Although CS cells (and indeed patients) are highly sensitive to UV, they exhibit normal DNA repair across the genome and patients have several distinct and arguably more extreme phenotypes than those with mutations in other NER factors, including neurodegeneration and many features reminiscent of premature ageing [8,9]. Repair defects in CS cells were found to be confined to transcriptionally active genomic regions. Patients with CS most commonly have genetic defects in two genes, *ERCC8* (encoding Cockayne Syndrome Protein A (CSA)) and *ERCC6*, encoding Cockayne Syndrome Protein B (CSB) [10]. It is known that CSB acts to recognise stalled RNA Polymerase II (RNAPII), and also acts to recruit downstream repair factors, with CSA residing in an E3 ubiquitin ligase complex, which ubiquitinates RNAPII and UVSSA to initiate TC-NER [11]. UVSSA was discovered more recently, in 2012, and mutations in the *UVSSA* gene cause a rare disorder, UV sensitivity syndrome (UVSS) and patients have a milder phenotype than that observed in XP and CS (with no increased cancer susceptibility, neurodegeneration or premature ageing) [12].

**Fig. 1:**
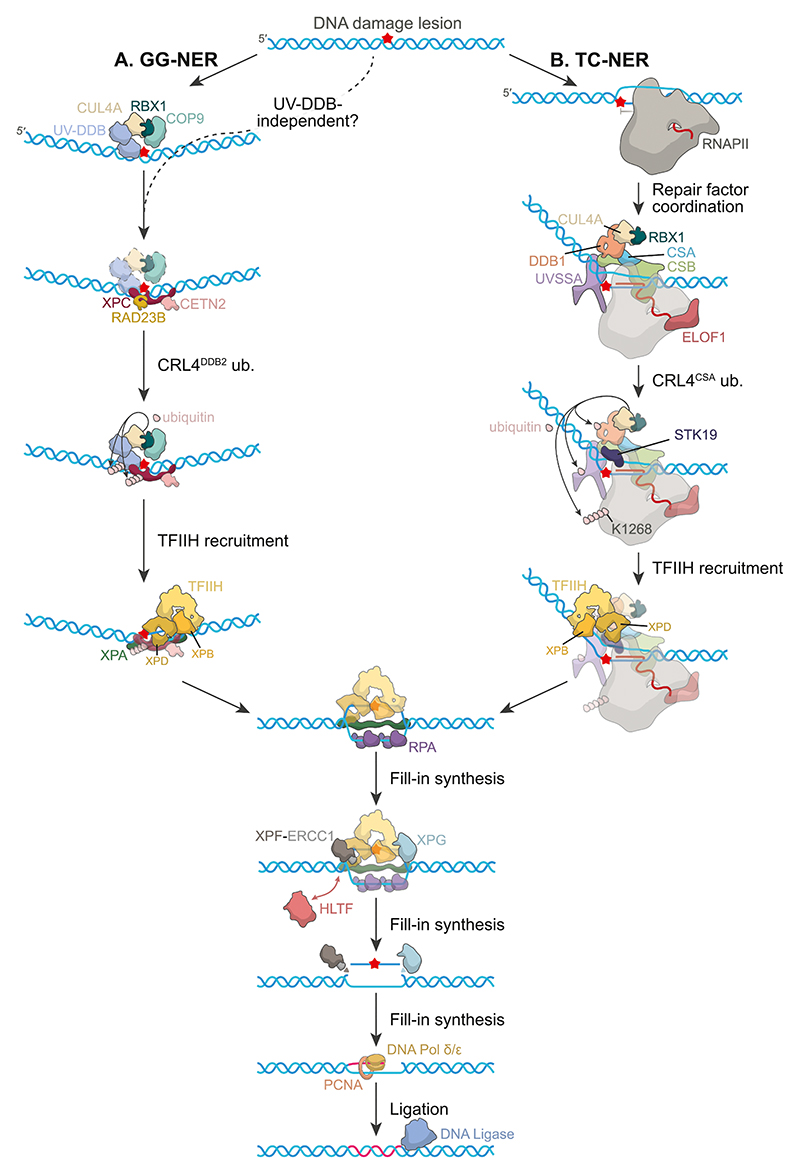
An overview of the major mammalian nucleotide excision repair pathways. A. For the repair of bulky (6−4) pyrimidine–pyrimidone photoproducts (PPs) and cyclobutane pyrimidine dimers (CPDs) in GG-NER, the initial recognition event can be UV-DDB-dependent or XPC-dependent. UV-DDB-dependent repair is more efficient and is initiated by UV-DDB binding directly to the DNA damage lesion. UV-DDB physically manipulates the duplex to ‘flip out’ the bulky base, providing a preferred binding site for XPC, which is in complex with Rad23B and CETN2. UV-DDB resides in a ubiquitin E3 ligase complex consisting of CUL4A, Roc1 and the COP9 signalosome. The UV-DDB-E3 complex then ubiquitinates XPC and UV-DDB. TFIIH is then recruited via interaction between the XPB subunit and XPC, and XPC and UV-DDB-E3 are released. For XPC-dependent repair, the XPC-containing complex recognises the lesion which then recruits TFIIH with interaction between XPC and the p62 subunit of TFIIH. The downstream steps of TC- and GG-NER are consistent after TFIIH recruitment and release of the lesion recognition factors. Next, the scaffolding proteins, XPA and RPA, are recruited, releasing the CAK subunit of TFIIH. This also allows for positioning of the repair endonucleases, XPF-ERCC1 and XPG. XPF-ERCC1 cuts first, 5′ to the lesion, followed by XPG at the 3′ side. This results in excision of the DNA damage lesion within a piece of ssDNA. Release of the DNA and repair factors is mediated by the translocase protein, HLTF. This allows for loading of PCNA and DNA polymerase δ or ε for gap filling synthesis, and RPA remains bound. After synthesis, the nick is sealed by DNA ligase I or III. **B**. TC-NER is initiated by the impediment of RNAPII progression by a bulky DNA lesion. The transient interaction between the transcription regulator protein CSB and RNAPII becomes stabilised, and the CSA-CUL4A-DDB1-RBX1 (CRL4^CSA^) ubiquitin ligase complex is recruited. The precise molecular coordination of the following steps is unclear, however, it is known that (i) the repair factor ELOF1 helps direct the ubiquitination of RNAPII subunit RPB1 at residue K1268 by CRL4^CSA^, (ii) UVSSA, a scaffold protein, helps to stabilise the complex and aids recruitment of TFIIH, and (iii) STK19 stabilises the repair complex by binding CSA, UVSSA and RPB1, aids UVSSA and RPB1-K1268 ubiquitination, and downstream loading of TFIIH via an interaction with the XPD subunit. Whether RNAPII and other core factors (i.e., CSB, CRL4^CSA^, STK19, ELOF1, UVSSA) are present after TFIIH recruitment is still unclear, although RNAPII is thought to be backtracked or released, and has been removed for clarity.

The initiation and damage removal events in TC-NER differ from the GG-NER pathway, although downstream incision factors are consistent. Our current understanding of the TC- and GG-NER pathways is summarised in Fig. 1.

However, the extreme and partly distinct clinical features of CS compared with XP and UVSS (i.e., primarily premature ageing versus sunlight sensitivity phenotypes) have suggested additional roles for the CSA and CSB proteins, outside NER, including in the rescue of general transcriptional defects and in the repair of other forms of DNA damage [8,13–16]. Additionally, mutations in other NER repair factors, XPG, XPB and XPD and XPF-ERCC1 manifest a small number of CS and XP/CS patients, raising questions about how and when RNAPII is removed during TCR [10,17–19].

This review will detail evidence to date of the TCR of DNA interstrand crosslinks (ICLs) and DNA-protein crosslinks (DPCs). Although beyond the scope of the current review, it is also important to acknowledge that evidence for transcription-coupling of other DNA repair pathways, in particular base excision repair, have been presented [20].

## 2 Crosslink damage as a contribution to CS phenotypes

Although CS patients have sensitivity to UV, this form of damage likely does not fully account for their observed clinical presentation, with endogenous forms of DNA damage also expected to inflict transcription-blocking lesions (TBLs). To help establish the metabolite which causes the damage underlying CS, researchers turned to mouse models [21]. Interestingly, knockout of CSA or CSB in mice does not recapitulate CS phenotypes. However, since CS patient mutations are often missense or frameshift mutations in CSA or CSB (resulting in premature truncations rather than absence of the protein), it is thought that patient phenotypes might sometimes reflect persistent repair intermediate complexes that cannot be displaced or removed. Therefore, when CSA and CSB are not present, TCR does not occur and prolonged stalling of RNAPII at the lesion may occur [22]. This causes alterations in the availability and regulation of the RNAPII pool and failures in transcription recovery [23].

Formaldehyde has emerged as a significant source of endogenous DNA damage, forming various damage lesions, including ICLs, DPCs and monoadducts [24]. Initial studies with mouse embryonic fibroblasts deficient in TC-NER (*Csb*^*m/m*^), GG-NER (*Xpc*^*-/-*^) or all NER (*Xpa*^*-/-*^) in combination with a lack of formaldehyde detoxification (*Adh5*^*-/-*^), indicated that TC-NER was crucial to protect against formaldehyde-induced DNA damage. These results were confirmed using the same knockouts in mice, with *Adh5*^*-/-*^ *Csb*^*m/m*^ mice exhibiting features resembling CS, including cachexia, kidney failure and growth defects. Additional methanol (which can be coverted into formaldehyde in a cellular context) exposure induced CS features in aged *Adh5*^*-/-*^ *Xpa*^*-/-*^ mice. Knockout of *Aldh2*^*-/-*^, which preferably catalyses oxidation of acetaldehyde (versus Adh5 which oxidises formaldehyde) in *Csb*^*m/m*^ mice did not result in CS phenotypes. This confirmed that formaldehyde, rather than acetaldehyde, was the responsible metabolite for the observed phenotypes [21].

## 3 Crosslinked DNA damage interferes with transcription

Some forms of DNA damage can be bypassed by RNAPII, such as certain types of oxidised base damage, although this can lead to the incorporation of a mismatched nucleotide [25], causing potential mutations or a requirement for downstream repair. When RNAPII does stall at a TBL, this is the initiation point for downstream repair. Among the DNA damage lesions that block RNAPII are those that induce significant duplex distortion [25,26]. Both ICL and DPC damage are known to induce duplex distortions and have subsequently been shown to stall RNAPII [27–29].

ICLs form a covalent linkage between the two strands of the DNA duplex, preventing strand separation. This is essential for transcription to progress and thus represents a blockage to fundamental cellular processes. DPCs, another form of covalently bonded DNA damage, are formed between DNA and proteins and result in a bulky lesion that is incompatible with transcription progression. Both types of damage are caused by bifunctional alkylating agents, i.e., a metabolite with two reactive nucleophiles. The causative endogenous and exogenous agents can be chemotherapeutic agents, metabolic by-products, and toxic environmental metabolites [30–34]. Both forms of damage are highly structurally diverse, with different perturbations to the DNA duplex structure. It is thought that the degree of helix distortion will cause RNAPII to stall at different distances from the damage site, although investigations to this effect are limited.

Regardless of the form of DNA damage, it is known that the transcription regulator ATPase protein, CSB, is fundamental to the initiation of all TCR. CSB interacts transiently with RNAPII during transcription, monitoring deviations in this process, and even acts to translocate RNAPII forwards [35,36]. When RNAPII stalls at damage, the CSB: RNAPII interaction becomes stable and the recruitment of downstream repair factors is initiated [37]. Without CSB, no known form of TCR occurs, and stalled TCR complexes accumulate, with implications for the transcriptional pool [22,23]. The differential processing of RNAPII at damage sites is correlated with the severity of TCR syndrome patient phenotypes [22]. This is demonstrated by experiments showing that RNAPII is cleared from damage in UVSSA KO cells, but not in CSA and CSB KO cells. This means that in CSA/CSB KO cells, the damage is shielded by RNAPII and cannot be efficiently repaired by other mechanisms, for example, GG-NER [22].

## 4 TC-ICL repair

### 4.1 Evidence for preferential repair of ICLs in active genes

ICL-inducing agents are one of the oldest and most widely used forms of chemotherapy, with studies of ICL repair mechanisms gathering significant research attention to uncover mechanisms of resistance to these drugs [38]. Initial studies of mammalian cell cycle phase dependence in ICL repair suggested that repair was confined to S-phase. This finding was based upon BrdU incorporation assays, however, this type of analysis can mask minor repair pathways [39]. Replication-coupled ICL repair (also called the Fanconi Anaemia (FA) pathway) is known to dominate in S-phase. Mutations in key repair factors in the FA pathway cause a rare genetic disease, FA, which manifests in impaired development, cancer predisposition, bone marrow failure and an extreme hypersensitivity to crosslinking agents [40]. However, early studies in bacteria and yeast demonstrated that in these organisms, modified NER-like pathways are required for ICL repair, including in non-dividing (non-replicating) cells, prompting investigation into whether similar pathways also operate in mammals [41,42].

Initial work on ICL repair employing *in vitro* transcription assays using HeLa cell extracts and immobilised DNA templates with site-specific damage lesions incorporated *via* triple-helix formation highlighted the differences in RNAPII fate depending on the type of adduct. Incorporation of a site-specific psoralen adduct (formed by UV irradiation of a psoralen-containing strand that anneals to the template strand) resulted in arrest of transcription elongation at the triplex site. Probes designed to generate different types of psoralen lesions demonstrated that ICL adducts arrested elongation whereas monoadducts terminate transcription. Immunoblotting experiments showed that arrested RNA-PII complexes contained RNAPII with increased phosphorylation (RNAPIIo, where phosphorylation occurs at the C-terminal heptapeptide repeats on the RBP1 subunit), suggesting elongation was stalled. The RNA transcript length suggested that RNAPII is arrested 1–2 nt before the adduct site [43]. However, the effect of triplex-formed psoralen adducts versus native psoralen adducts on transcription and duplex structure is likely to differ significantly.

Work conducted in the Hanawalt laboratory provided the first cellular evidence for mammalian ICL repair being targeted to active genes. Many of these initial experiments quantify preferential repair of monoadducts versus ICLs in active genes compared to the global genome, using techniques generally involving Southern hybridisation of DNA that is denatured (to separate damaged and undamaged DNA fragments) then renatured and analysed on native gels. Initial work studying the repair of psoralen adducts in the dihydrofolate reductase (DHFR) gene in human fibroblasts showed that ICLs were repaired preferentially to monoadducts in this gene (80 % of ICLs versus 45 % of monoadducts, over 24 h) [44]. This was the first demonstration that the TCR response varies depending on the lesion. A follow-up study showed that psoralen-induced ICL damage was removed more readily in active genes rather than in the bulk genome (90 % versus 31–36 %), again implicating transcriptional status in the ICL repair response [45]. Generally, more damage was observed in active genes compared with the bulk, likely attributed to sequence and chromatin structure; psoralens are more reactive with linker DNA (i.e., the DNA between nucleosomes) and transcribed regions have a more open chromatin structure, so the bases are more exposed to damage.

To study ICL repair in G_0_/G_1_ (thus in the absence of replication-coupled repair, which is the dominant ICL repair pathway), CHO cells emerged as a useful research tool as they can be held in G_1_ indefinitely, simply through isoleucine starvation, then readily released back to a cycling state by re-introducing isoleucine. Using this system and cisplatin as the damaging agent (which forms both ICLs and mono-adducts, accounting for 5 % and 95 % of lesions, respectively), ICL damage was preferentially repaired in active genes, however at very high levels of crosslinking, this discrepancy between genomic regions was lost [46,47]. Differences in the repair rate were also noted between cisplatin and previous studies of psoralen ICL repair. Preferential formation and repair of nitrogen mustard-induced ICLs in active genes has also been observed, again with a dose-dependent response [46,47]. Further studies in CHO cells provided more insight into the importance of TC-ICL repair for preventing mutagenesis. Although psoralens can cause both monoadducts and ICLs, this is dictated by DNA sequence; 5′TpA sites are more susceptible to ICLs, whereas monoadducts are more readily formed at 5′ApT sites. Indeed, after psoralen damage, mutations in the *APRT* gene were primarily located at 5′TpA sites [48].

### 4.2 The role of modified TC-NER in repairing ICLs

Subsequently, the identities of TCR factors were emerging, primarily based upon both patient and *in vitro* studies. Complementation studies in CHO cells defective in the NER factor ERCC1 indicated that this gene is required for ICL unhooking (and likely repair) in actively transcribed genes. ERCC1-deficient cells had significant cisplatin sensitivity that could be rescued by ectopic expression. ERCC1-dependent repair was also shown to favour ICLs over monoadducts [49]. However, repair was not compared to that in the bulk genome, although a prior complementation study in HeLa cells also indicated a more pronounced effect of ERCC1 transfection in rescuing sensitivities to mitomycin C (MMC) induced ICL damage than UV-induced damage [50].

Biochemical studies using mammalian cell extracts (WT or NER-factor deficient) on psoralen ICL-containing dsDNA noted that dual incisions are made 5′ to the ICL on both strands, but there is no incision 3′ to the ICL. These incisions required the core NER factors XPF-ERCC1, XPD and XPG to be present and result in a 22–26 nt gap [51].

Wang *et al*. provided subsequent insight into the role of NER factors in recombination independent ICL repair in mammalian cells [52]. Cell lines defective in homologous recombination (HR) (XRCC2- or XRCC3-deficient) or NER factors (XPA- or ERCC1-deficient) were generated, and by survival assay, it was shown that an NER-dependent and HR-independent ICL repair pathway was operating. Further NER factors, XPA, XPF-ERCC1, TFIIH and XPG, were also shown to participate in this pathway by a plasmid reporter assay (with a psoralen ICL lesion placed between a promoter and a fluorescent reporter to study repair of an active gene) using cell lines deficient in individual repair factors. No impact on repair was noted in XPC-deficient cell lines, although preferential repair on the transcribed strand was observed, implicating that TCR rather than GG-NER dominated. Repair was also shown to be error-prone, using an ICL placed in a restriction enzyme site and studying its cleavage post-repair [52]. A follow-up study also by the Li group used an analogous reporter assay with a single, site-specific psoralen or MMC ICL lesion. Cell lines defective in CSA or CSB were shown to be ICL repair deficient, although not to the same extent as cells with an XPA or ERCC1 deficiency [53]. Subsequent work using a similar plasmid-based reporter assay, with a plasmid treated globally with MMC, also implicated an NER factor-dependent ICL repair pathway separate from GG-NER, with XPA-deficient but not XPC-deficient cell lines showing ICL repair defects [54].

Smeaton *et al*. corroborated previous observations that in NER-dependent ICL repair, dual incisions are made 5′ to the site of the lesion (employing substrates harbouring synthetic N4C-ethyl-N4C ICLs). This was shown to require XPF-ERCC1 [28]. Studies by Muniandy *et al*. in 2009, however, showed that in G_1_ (i.e., in the absence of efficient homologous recombination) that an XPC-dependent, NER-dependent (i. e., modified GG-NER *versus* TC-NER initiation) pathway operates to remove laser-induced psoralen ICLs. Therefore, in many studies, delineating these pathways is not possible and makes interrogation of the TC-ICL repair pathway in isolation challenging. XPC-dependent ICL repair is also likely to be lesion structure dependent, based on the mechanism of XPC damage by monitoring duplex distortion followed by the formation of a damage-stabilised complex [55].

In 2012, further work using promoter-based reporter assays to study the repair of cisplatin ICLs indicated a role for the TC-NER proteins, CSB, XPA and XPG (and not XPC) in repair and the translesion synthesis proteins, Rev1 and Polξ, in error-free bypass of the ICL. Interestingly, under these conditions, with cisplatin monoadducts, CSB- and XPC-deficient cell lines did not exhibit impaired repair, suggesting the specific role of TC-NER proteins in the repair of cisplatin-induced ICLs. Depletion of proteins involved in HR, the FA pathway and mismatch repair (MMR) did not affect NER-dependent ICL repair, again delineating this as an independent pathway. CSB- and XPA-deficient human fibroblasts were also shown to be sensitive to MMC and further experiments considered replication-independent repair in isolation (by synchronising cells in G_0_/G_1_). Here, WT and CSB-deficient cells were arrested in G_0_/G_1_ and treated with MMC, then released into S-phase after repair had occurred. After release, both cell lines accumulated in G_2_/M after 24 h, however, CSB-deficient cells remained in G_2_/M, suggesting that more ICLs were present upon entering S-phase. By depleting FANCD2 in CSB-deficient and corrected cell lines, and studying ICL sensitivity, replication-dependent and TC-NER factor-dependent ICL repair were shown to act in parallel, with both contributing to the cell survival response [56]. Contrary to prior studies, repair was in fact shown to be error-free (again by including the ICL inside a restriction enzyme recognition sequence).

However, although these studies demonstrated the presence of a replication-independent ICL repair pathway targeted to active genes and conducted by TC-NER proteins, coupling with the transcription apparatus had not yet been directly demonstrated, nor had the molecular details of how the ICL could be removed from both strands of the duplex been elucidated. Studies conducted in the Wilson laboratory took a more exploratory approach, starting with the discovery of new interaction partners of CSB by yeast two-hybrid (Y2H) screen [57]. The second to top hit was *DCLRE1A*, the gene encoding SNM1A, a 5′-to-3′ DNA exonuclease with roles in ICL repair, whose homolog in budding yeast, Pso2, has a well-established role in ICL repair in both dividing and non-dividing cells [57]. SNM1A is a metallo-β-lactamase (MBL) fold-containing protein with an inserted β-CASP domain (which confers nucleic acid binding). Y2H studies also identified that the C-terminal domain of CSB (amino acids (a.a.) 1187–1493) and the C-terminal domain of SNM1A (MBL and β-CASP domains, a.a. 608–1040, referred to as ΔN-SNM1A) were the key interacting domains. This interaction was further validated by immunoprecipitation experiments. Interestingly, although the presence of ΔN-SNM1A did not affect the ATPase activity of CSB, the presence of CSB was shown to enhance the nuclease activity of ΔN-SNM1A on both single-stranded (ss) and double-stranded (ds) undamaged DNA. Kinetic analysis attributed this stimulation of SNM1A nuclease activity by CSB on ssDNA to the physical interaction between the two proteins, increasing the affinity of SNM1A for DNA, with CSB binding to ssDNA not experimentally proven at this point. To study the recruitment of CSB and SNM1A to ICL damage, fluorescently tagged fusion proteins were studied by confocal microscopy with damage induced by laser irradiation. Both SNM1A and CSB were independently shown to recruit to ICL damage, and this recruitment was reduced if cells were treated with an RNAPII inhibitor, α-amanitin. Studies with both CSB and SNM1A showed co-localisation at ICL damage sites, and in CSB-deficient cells, SNM1A accumulation was reduced. Studies of replicating versus non-replicating cells with stable CSB knockdown showed that in both cycling states, CSB deficiency resulted in sensitivities to monoadducts and ICLs, with a more pronounced effect with ICL damage. Comet assays also showed deficiencies in ICL unhooking in non-dividing CSB-deficient cells [57]. Follow-up recruitment studies of CSB to different types of DNA damage showed that CSB recruitment was faster and more sustained at ICLs than monoadducts, double-stranded breaks, and oxidative damage and that this accumulation was reduced in the presence of a transcription inhibitor. The C-terminal domain of CSB (particularly the ubiquitin-binding domain) was also shown to be crucial for recruitment [58].

**Fig. 2:**
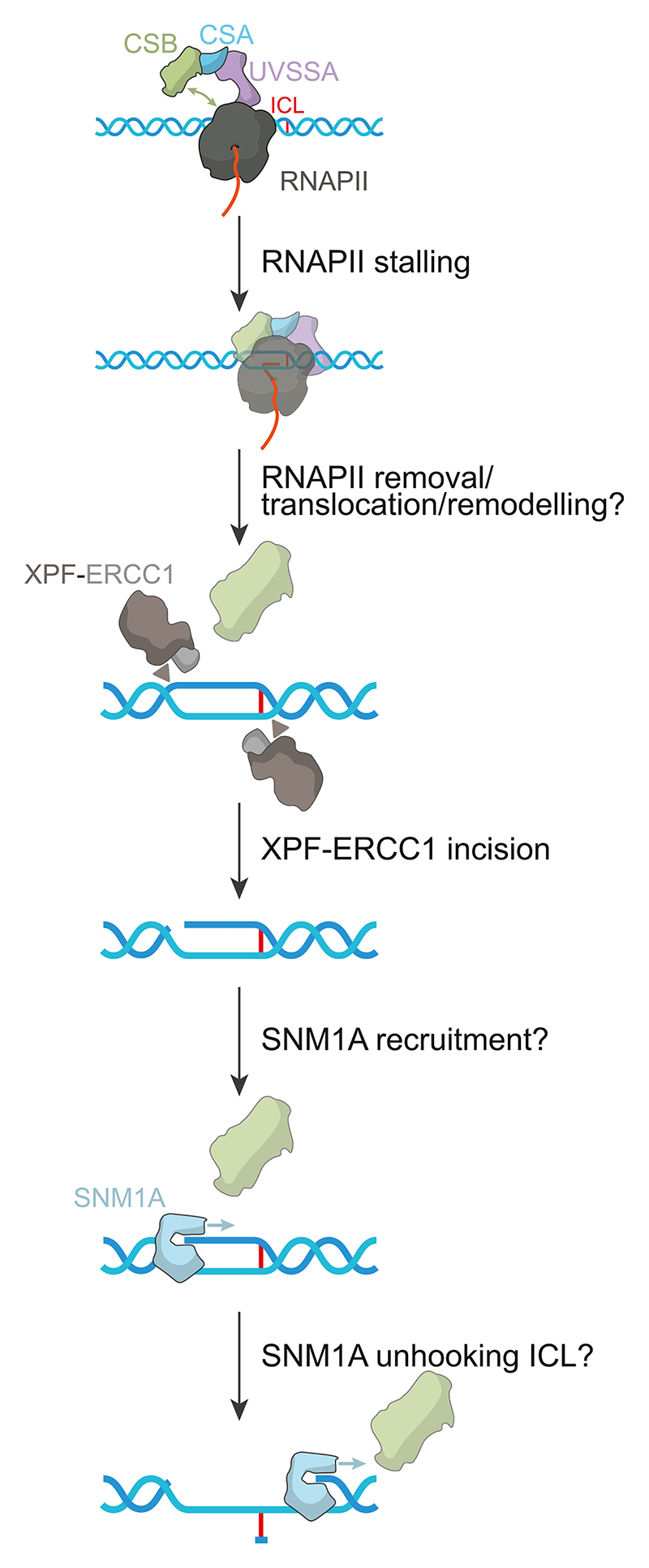
Current understanding of TC-ICL repair. Consistent with TC-NER, RNAPII stalling at the damaged lesion initiates downstream repair, with the interaction between CSB and RNAPII becoming sustained. TFIIH and other repair factors (e.g., XPA, RPA) likely coordinate duplex distortion to allow for lesion excision, but since this remains to be determined, they are omitted for simplicity. XPF-ERCC1 is required for ‘unhooking’ the ICL by cutting the junction of the bubble 5′ to the ICL. There are two possible locations for XPF-ERCC1 incision which are shown, but it is unknown which side is cut in vivo. This generates a 5′ phosphate, which is a requirement for nuclease activity by the exonuclease, SNM1A, which processively hydrolyses the DNA through the ICL, which is likely mediated by CSB. The presence of RNAPII during lesion excision is unclear, and it is also unknown how SNM1A digestion is terminated. Excision factors are then released by an unknown mechanism. Gap filling and ligation are thought to occur in an analogous manner to traditional NER.

Subsequently, studies in the Gautier lab provided further insight into the identities of other repair factors involved in TC-ICL repair [59]. Cells were shown to be sensitised to an ICL-inducing agent (cisplatin) with UVSSA knockout, with the phenotype rescued by complementation with FLAG-UVSSA. Expression of a TFIIH-binding deficient UVSSA mutant also showed cisplatin-sensitivity, suggesting a role of the TFIIH-UVSSA interaction in repair. A plasmid-based reporter assay was used to study TC-ICL repair in isolation, incorporating a single SJG-136 induced ICL lesion inserted between a strong, CMV promoter and a reporter gene. Again, repair deficiencies were observed with defective UVSSA-TFIIH binding and UVSSA knockout. Additionally, IP-MS studies showed the presence of SJG-136-induced interactions between UVSSA, and CSA, CSB and TFIIH components, and colocalisation studies in the presence/absence of α-amanitin (a molecule that blocks RNAPII elongation) demonstrated that formation of the repair complex was transcription-dependent. However, both SJG-136 and cisplatin do not induce only ICL damage, with intrastrand crosslinks and monoadduct lesions also formed. Our current understanding of TC-ICL repair is summarised in Fig. 2.

### 4.3 Insights into DPC repair

In contrast to TC-ICL repair, until relatively recently, there has been significantly less research attention on how DPCs are repaired, let alone in the context of transcription [60]. The types of lesions that can form are highly diverse, dependent on both the chemical nature of the linkage and the identity of the bound protein [61,62].

Initial reports that DPCs can block transcription were reported in bacterial systems. Briefly, it was shown that covalently-trapped Topoisomerase I complexes (TOP1cc) on the template strand can block transcription, causing premature transcription termination [63]. *In vitro* transcription assays and DNA footprinting assays using bacteriophage SP6 RNA polymerase showed that the RNA polymerase halts 10 bp away from the nucleoside to which TOP1 is bound, corresponding to the border of where TOP1 occludes DNA. This effect was only observed on the template strand [63]. Subsequent studies with T7 RNA polymerase corroborated these results and demonstrated that TOP1cc causes ss breaks on the template strand after collision with RNA polymerase [64].

Historically, mammalian TC-DPC investigations have primarily focused on the irreversible covalent linkage of the DNA-modifying enzyme Topoisomerase I (TOP1), to DNA by the anticancer drug, camptothecin (CPT) due to its clinical relevance [65]. However, many other proteins can be covalently trapped on DNA, both enzymatically and non-enzymatically [33]. Repair generally requires the activity of specific proteases to remove the covalently bound protein or factors that can excise the covalent linkage [34,60].

The first suggestion of the role of CS proteins in DPC resolution was noted in 1993 by the Johnson laboratory [66]. CS patient fibroblasts were noted to be highly sensitive to CPT, with the toxicity likely indicative of the lack of resolution of dsDNA breaks formed during defective repair. Other studies indeed demonstrated that TOP1-DNA covalent complexes (TOP1cc) do arrest transcription in mammalian cells [63,64, 67], with the requirement for TOP1 proteasomal degradation downstream in repair [68,69]. By immunoprecipitation experiments, it was shown that in the presence of transcription inhibitors, TOP1cc resolution was reduced [68,69]. Treatment with both CPT and a 26S proteosome inhibitor (MG132) was shown to block transcription recovery, by uridine incorporation experiments. RNAPII was also phosphorylated and degraded in response to CPT treatment, suggesting the role of the 26S proteosome in degrading both RNAPII and TOP1cc in a TC pathway [68]. Notably, the presence of a DNA polymerase inhibitor, aphidicolin, did not affect degradation, suggesting that TOP1cc complexes are removed in a TC manner. It was later found that in the presence of TOP1cc, RNAPII phosphorylation occurs specifically on Serine-5 in the heptad repeats on the major RNAPII subunit, Rpb-1 C-domain, mediated by CDK7 (determined by using specific antibodies and kinase inhibitors) [70]. The authors also considered the role of BRCA1, a DNA repair protein implicated in homologous recombination, as it was known at this stage to associate with transcribing RNAPII and is an E3 ligase [71]. Experiments in *Brca1*^+*/*+^, *Brca1*^*-/-*^ and human BRCA1 complemented *Brca*^*-/-*^ mouse embryonic fibroblasts (MEFs) implicated BRCA1 in TOP1cc degradation but not RNAPII degradation [70].

The role of CS proteins in DPC repair was further highlighted by studies of CS patient cell lines, which express truncated CSB or CSB-piggyBac protein (CSBpB). CSBpB is a form of CSB with the piggyBac transposable element 3 integrated into CSB exon 5 and is produced alongside wild-type CSB in healthy individuals, although CSB outcompetes CSBpB, so it is not deleterious [72]. However, in some CS patient mutations, wild-type CSB is truncated and the CSBpB fusion protein becomes functionally relevant. The presence of CSBpB was shown to impede CSB’s ability to aid TOP1cc repair, overall implicating CSB in DPC repair.

DPC damage is also inflicted by the anti-cancer drug, 5-Aza-2′-deoxycytidine (5azadC), which has a dual function in inhibiting DNA methylation and covalently trapping DNA methyltransferases (DNMTs) on DNA. Work by the Orta laboratory in 2018 highlighted the crucial role of CSB in repairing DNMT1-DPCs [73]. Studies were performed measuring chromosomal aberrations as markers of ds breaks, using a CS patient cell line, complemented with CSB fused to GFP. CSB foci accumulated after 5azadC treatment, co-localising with γ-H2AX and DNMT1-GFP foci. Differences in RNA synthesis were studied, with decreased transcription rates observed in CS patient cells post-5azadC treatment, suggesting that CSB is important for repair signalling. Repair of trapped DNMT1 was also reduced in CS patient cells, and by monitoring cell survival, levels of repair foci or micronuclei induction, with treatment of 5azadC and the presence/absence of a transcription inhibitor indicated that the pathway was indeed coupled to transcription [73].

At this stage, the requirement for CSB in TC-DPC repair was well-established, although the requirement for other TC-NER factors was unclear. Plasmid reporter assays, with site-specific DPC lesions, were carried out in human or CHO cells deficient in different NER or base excision repair (BER, the pathway acting to remove oxidative DNA damage lesions) factors to study their repair capabilities. Deficiencies in NER factors XPA or XPD reduced repair capacities, and repair was favoured on the template strand, overall suggesting roles for TC-NER in repair. Repair was also more efficient when smaller proteins were covalently bound (4 kDa *versus* 36 kDa), likely reflecting DNA accessibility.

Over the past few years, the rapid advancement of sequencing technologies has allowed for the precise determination of DNA damage lesion location on a genome-wide level [74]. In 2024, three back-to-back papers studied TC-DPC repair using these methodologies [75–77], and their findings have been reviewed previously [78,79]. DPC-seq was developed to monitor DPCs across the genome by selectively isolating protein-crosslinked regions of genomic DNA, followed by protein degradation and next-generation sequencing. This, in combination with CRISPR interference screens in the absence/presence of formaldehyde (to induce DPC formation), allowed for understanding of the fate of RNAPII post-DPC stalling [75–77,79,80]. The cellular response to DPC damage and UV damage was compared. Common to both pathways, CSA and CSB are initially recruited. However, downstream, key NER factors (e.g., XPG and XPF-ERCC1) and repair factors ELOF1 and UVSSA were shown to be non-essential. Although RNAPII ubiquitination was observed upon formaldehyde and decitabine treatment (and this PTM is required for functional TC-NER), a defective mutation, RPB1^K1268R^, exhibited only mild repair defects. This further confirms that repair is excision-independent; in TC-NER, RPB1 ubiquitination at K1268 is required for TFIIH recruitment, which acts to unwind the DNA duplex, allowing for lesion removal through dual cuts by the nucleases, XPG and XPF-ERCC1 [81]. Crosslinked proteins were found to be targets for ubiquitination, likely by the CRL4^CSA^ complex, targeting them for proteasomal degradation. Downstream degradation of the DPC was shown to require VCP/p97, which acts to unfold proteins, preparing the DPC for proteasomal degradation. RNAPII and p97 were also shown to interact [75,76]. To restart transcription, proteasomal degradation of the transcription inhibitory factor ATF3 is induced [77]. Studies of CHO cell extracts with XPG knockout showed deficiencies in DPC repair, both in a plasmid reporter assay and in gel-based assays with DNA damage-containing oligonucleotides [82], however in more recent studies, the core NER factor, XPA, was shown to not be required for recovery of RNA synthesis after formaldehyde treatment [75,76,78]. Additionally, studies by the Schumacher laboratory in *C. elegans* demonstrated that multiple repair pathways contribute to formaldehyde resistance. CSB was found to be required for repair of formaldehyde-induced DPCs. Overall, formaldehyde-resistance was found to be NER-independent (with a minor role of XPA) during developmental growth, but NER-dependent in adulthood, suggesting different repair mechanisms operate during different stages of development [83]. This therefore raises questions about how the residual peptide is removed, although likely an excision repair pathway (BER or modified NER) is involved.

**Fig. 3:**
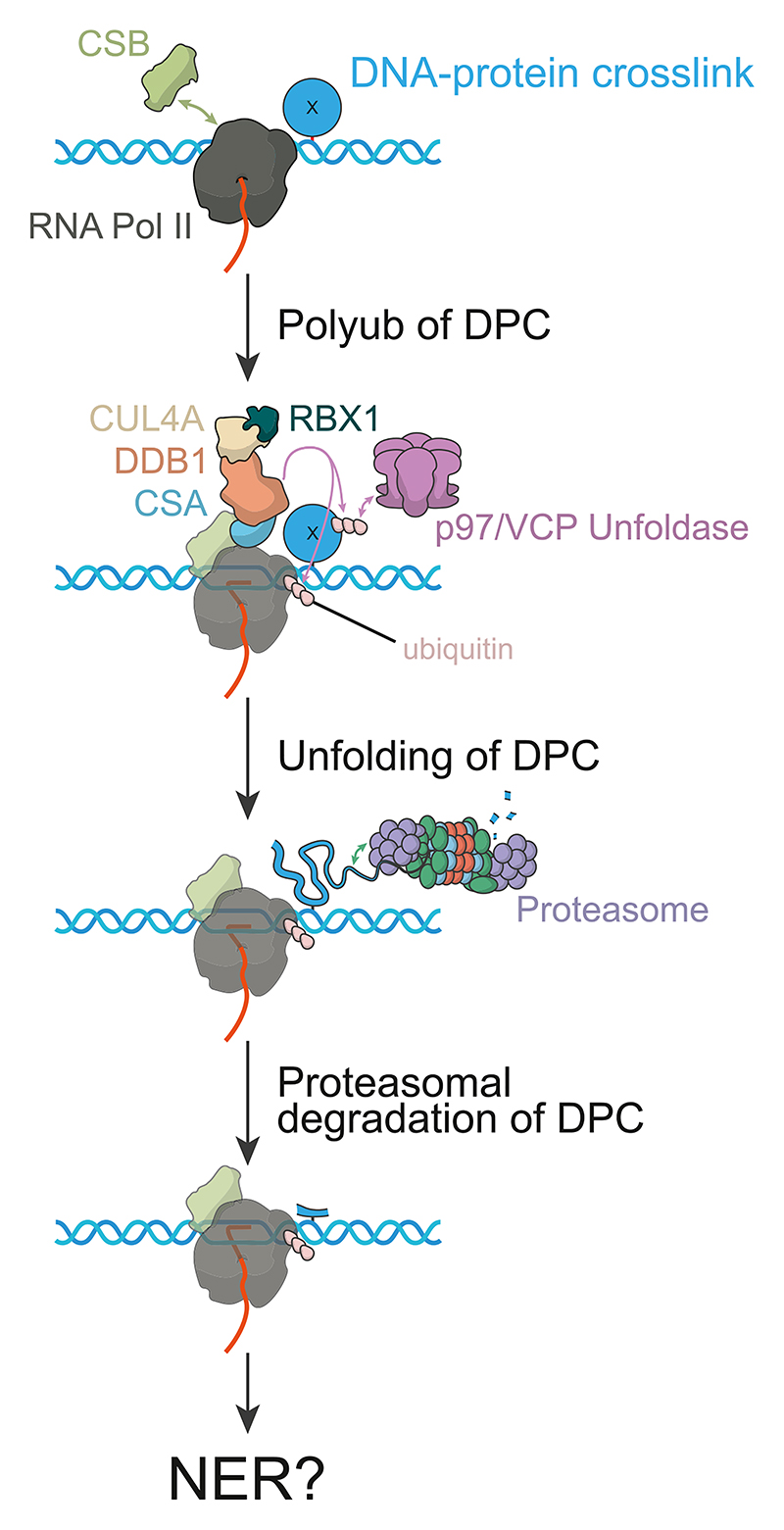
Model of TC-DPC repair. A DPC represents a fundamental blockage to RNAPII, resulting in transcription stalling. The interaction between CSB and RNAPII becomes sustained, and the CRL4^CSA^ complex is recruited. RNAPII (RPB1-K1268, although this is not functionally relevant) and the crosslinked protein are then ubiquitinated by the CRL4^CSA^ complex. TFIIH is potentially involved, aiding duplex manipulation to allow for DPC access. The unfoldase protein, p97, in complex with VCP, is then recruited to aid DPC degradation by the proteasome. It is thought that RNAPII is not evicted during DPC degradation. Degradation results in a crosslinked peptide remaining on the template strand, which would likely be a substrate for NER or another excision repair pathway [82,84].

In summary, studies of TC-DPC repair have highlighted that in stark contrast to TC-NER and TC-ICL repair, removal of the lesion does not require DNA resection and resynthesis. Instead, the TCR initiation complex and the crosslinked protein are ubiquitinated and targeted for degradation. The protease involved is unclear, however, the dedicated DPC protease, SPRTN, which is key to replication-coupled and GG-DPC repair, is not required. The model of TC-DPC repair to date is given in Fig. 3.

### 4.4 Clinical relevance of understanding transcription-coupled crosslink repair

Terminally differentiated, non-dividing cells predominate in adults and rely on replication-independent repair mechanisms (GG and TC) to remove DNA damage lesions and allow for active genes to be accurately transcribed [85].

Crosslinked DNA is the most deleterious form of damage as it represents a fundamental, steric blockage to DNA processing machineries. ICL-inducing agents are important as therapeutics (most notably in cancer) and are created through the metabolism of alcohol, making their repair highly relevant to the general population. Resistance to crosslink-inducing chemotherapeutics is a common evolutionary mechanism in cancer and is often attributed to enhanced or upregulated DNA repair [38,86]. Notably, CSB-deficient cells are more sensitive to crosslinking agents when treated in G_0_/G_1_[56]. Another potential avenue for research is the role of TCR in stem-like cancer cells, which are a subpopulation of cancer cells that often persist after chemotherapy, and can lead to cancer relapse [87]. These cells rarely cycle and are reported to possess enhanced DNA repair, so evade the effects of therapies that invoke DNA damage by targeting mitosis. GG and TCR pathways are therefore more heavily relied upon than in normal cells to remove DNA damage, and so targeted therapies could be a way to selectively kill these cells [88]. Overall, understanding how damage from crosslink-inducing chemotherapeutics is repaired will help develop new, improved therapies, understand mechanisms of resistance, and potentially provide routes to systemically sensitise patients and overall enhance crosslink therapy effectiveness [38,89,90].

Defective TCR is also closely linked to both physiological ageing and disorders that clinically present as premature ageing. Recent studies have shown that the accumulation of TBLs with age, caused by endogenous DNA damage, is responsible for increased RNA polymerase stalling and thus changes in gene expression [91–93]. These overall changes in the transcriptome will lead to the hallmarks of ageing [94]. We also know from mouse studies that the crosslinking agent, formaldehyde, is the endogenous metabolite responsible for a rare premature ageing disorder, CS, although a direct link with physiological ageing has not been shown [21].

## 5 Conclusions and future perspectives

Although studies of TCR have focused on repair of bulky adducts by the NER pathway, particularly UV-light induced photodimers, other forms of DNA damage can block transcription. ICLs and DPCs represent a fundamental blockage to RNAPII progression and the implications of such crosslinked damage in ageing and chemotherapy resistance have extended research interest into these areas.

Insight into TC-DPC repair has been advanced greatly by recent studies [75,77,83,95]. Although lesion sensing employs the same repair factors as TC-NER, downstream repair differs drastically, instead involving the VCP/p97 unfoldase and degradation of the crosslinked protein by the proteasome. The TC-DPC repair pathway varies significantly from TC-NER, which instead uses a host of repair factors (NER factors) to excise and replace the damaged DNA. In contrast, our understanding of TC-ICL repair is significantly more incomplete and our molecular insights are confined to studies of interaction and DNA-damage recruitment between CSB and the ICL-processing nuclease, SNM1A [57]. Other NER factors, including XPF-ERCC1, TFIIH and XPA, are likely involved (although the role of XPG is inconclusive). The involvement of SNM1A also suggests that a substantially different mechanism to TC-NER is required.

With both types of damage, key questions remain around the mechanism of RNAPII stalling, pathway regulation and RNAPII fate after repair. In TC-DPC repair, further work is needed to understand (i) how the nature of the crosslink and the crosslinked protein alters the mechanism of repair, (ii) how downstream degradation from the proteasome is coordinated, and (iii) how the crosslinked peptide remaining after proteasomal degradation is removed. We understand even less about TC-ICL repair, with fundamental questions arising, including the identities of repair factors, the fate of SNM1A after ICL processing and the structural and molecular details of repair.

Studying TCR in isolation is challenging, and particularly with crosslink repair, there is significant redundancy with replication-coupled and GG pathways. The rise of sequencing technologies that can specifically detect locations of DNA damage and repair on a genomewide level, such as SAR-seq [96], will be helpful to elucidate the role of TCR in all cell types. There is also a dearth of selective crosslinking agents, and RNAPII stalling and downstream pathway regulation are likely to differ depending on the specific lesion generated, particularly as different degrees of helix distortion/DNA occlusion may be induced. A focus on using recombinant pathway reconstitution compared with in-cell studies will likely hold the answers to these questions.

## Data Availability

No data was used for the research described in the article.
